# Psychometric properties of the Czech version of the brief Older People Quality of Life questionnaire (OPQoL-brief)

**DOI:** 10.1186/s12877-022-03198-x

**Published:** 2022-06-22

**Authors:** Radka Bužgová, Radka Kozáková, Renáta Zeleníková, Katka Bobčíková

**Affiliations:** Department of Nursing and Midwifery, Faculty of Medicine, University of Ostrava, Syllabova 19, 700 30 Ostrava, Czech Republic

**Keywords:** Elderly, Quality of life, Reliability, Validity

## Abstract

**Background:**

To evaluate the established interventions used for older adults, it is appropriate to use validated questionnaires for quality-of-life assessment. For older people, it is suitable to use specific questionnaires designed for old age and aging, with a lower number of questions. The aim of this research was to verify the psychometric properties of the Czech version of the OPQoL-brief questionnaire for seniors living in home environment in a community so that it can be used within the Czech Republic.

**Methods:**

A cross-sectional study was performed on older adults in the Moravian-Silesian Region living at home. The study included 954 senior citizens (≥ 65 years, cognitively intact) (without diagnosed dementia, able to sign an informed consent). To test the psychometric properties of the created questionnaire, we tested the validity (construct validity, discriminant validity, convergent validity) and reliability (internal consistency, test–retest reliability).

**Results:**

The single-factor model of the OPQoL-brief scale (CFI = 0.971, TLI = 0.959, RMSEA = 0.061, SRMR = 0.034, GFI = 0.960) was confirmed, for which excellent reliability was found (*α* = 0.921, ICC = 0.904). An inter-item correlation exceeding 0.5 was found for all items. Furthermore, a significant correlation was found between the overall score of OPQoL-brief and the scales measuring depression (*r* =  − 0.520; *p* < 0.001), anxiety (*r* =  − 0.355; *p* < 0.001), sense of coherence (*r* = 0.427; *p* < 0.001), and self-esteem (*r* = 0.428; *p* < 0.001).

**Conclusion:**

The results of our research revealed that the shorter Czech version of the OPQoL-brief questionnaire has appropriate reliability and validity and can be recommended for both health and social services to assess the quality of life of senior citizens in a community.

## Introduction

According to the data of the Czech Statistical Office, from 2001 to 2017, the age group of people 65 years old and older increased in number by 625,600 from 1,414,600 to 2,40,200. In 2017, the number of older adults in the Czech Republic exceeded 2 million for the first time in history. The aging of the population has thus become a current topic in the Czech Republic and is one of the priorities of applied health research [1].

According to the strategy of preparation for population aging, from 2019 to 2025 [2], supporting older adult health prevention and specialized geriatric care is an important priority. In this context, it is necessary to view health and illness in old age holistically so that the physical, mental, social, and spiritual difficulties are perceived in a complex way as a part of the overall quality of life (QoL) of the older adult. This way, their QoL may be improved as well as their active participation in society. QoL is considered a positive indicator of an individual's overall condition, which allows a comprehensive evaluation of the focus of health and psychosocial intervention [3].

To evaluate the effectiveness of any prevention measures and established interventions to maintain or improve the QoL in old age, it is necessary to use tools that can measure such construct. QoL is defined by the World Health Organization [4] as an individual’s perception of their position in life in relation to their objectives, expectations, standards, and concerns in the context of the culture and value system in which they live. HRQOL is described by the Centers for Disease Control and Prevention as “a person’s or a group’s perception of physical and mental health across time” [5]. Bowling et al. [6, 7] reported that the foundations of QoL emphasized by people aged 65 + were psychological wellbeing and positive outlook, health and functioning, social relationships, leisure activities, neighborhood resources, adequate financial circumstances, and independence.

Several instruments were developed to evaluate HRQoL using generic and specific questionnaires. While generic questionnaires have the advantage of being applicable to a wide range of populations and conditions, (e.g., SF-12 [8], SF-36 [9], EQ-5D [10], WHOQoL-BREF [11]), condition-specific instruments (focused on one particular health condition or illness) or population-specific tools (e.g., older person-specific) may be more sensitive and therefore more suitable for use within particular patient groups or populations. Brazier et al. [12] states that the EuroQoL and SF-12/SF-36 questionnaires can also be used for the elderly population. However, it should be emphasized that these questionnaires do not cover any areas exclusively important for seniors [3]. Aging can be considered a multifactorial process, and therefore, the tools that include the specifics of aging should be used in older adults. This is one of the reasons why, in 2009, the WHOQoL working group developed a questionnaire for the QoL of the elderly population WHOQoL-OLD [3]. Other questionnaires created for senior age include the CASP-19 [13], QUAL-E [14], EQOLI [15], and OPQOL [16]. CASP-19 was designed to cover the active and beneficial experiences of later life, rather than simply focusing on the medical and social care issues that had traditionally been seen to characterize any aging research [15].

The EQOLI and QUAL-E questionnaires focus on a specific topic: the QoL at the end of one’s life. Other questionnaires were developed as well, related to QoL, e.g., the Manageable Geriatric Assessment, designed by a European group of family doctors in Germany. The questionnaire allows for the efficient rapid screening of relevant problems related to possible loss of autonomy in the elderly [17, 18]. Furthermore, there is the Adult Social Care Outcomes Toolkit [19], which is primarily designed for social care assessment, not health care evaluation.

The WHOQoL-OLD, OPQOL, and CASP-19 questionnaires are most often used in the research of older adults living in a community. Bowling and Stenner [20] compared these three questionnaires and their suitability to be used in senior citizens. All three questionnaires performed well with the cross-sectional samples; however, only OPQoL met the criteria for internal consistency in the Ethnibus samples. The Czech versions of WHOQoL_OLD-26 items [3] and OPQoL_35 items [21] were developed. However, a shorter version of the questionnaire is not available for a faster assessment of the QoL of older adults in community care. Shorter versions of questionnaires are currently very popular and used in various population groups. Reducing the number of items simplifies the administration, shortens the time needed to complete the questionnaire, and increases the return ratio. For this reason, we decided to translate and validate a shorter version of the OPQoL-brief questionnaire, which contains 13 questions [6], as a part of our project aimed at supporting healthy aging in a community.

## Aims

The aim of this research was to describe the psychometric properties of the Czech version of the OPQoL-brief questionnaire in older adults living at home in a community. Another goal was to determine the validity of the single-factor scale model. Furthermore, the aim was to verify the reliability and validity of the scale for the Czech population of seniors in community care.

## Methods

### Study design and participants

A total of 954 older adults from the Moravian-Silesian Region who live in a home environment participated in the research. The criterion for inclusion in the research group was that the person had to be aged 65 or older, and they had to be cognitively intact (without diagnosed dementia, and, able to sign an informed consent form). The older adults were approached in all districts of the Moravian-Silesian Region through more than 10 organizations (e.g., seniors clubs, community centers), through libraries, and through the Center for Prevention and Support of Healthy Aging of the Faculty of Medicine, University of Ostrava. The questionnaires were distributed to the participants in both printed and electronic form. According to data from the Czech Statistical Office from 2021, approximately 236,000 people over 65 years of age live in the Moravian-Silesian Region. Our sample included 0.4% of these seniors.

### Instrument

To evaluate the QoL, we chose the OPQoL-brief questionnaire [6], which is the short version of the OPQoL-35 and which measures the QoL of people over 65 years of age. The OPQOL-35 questionnaire was developed by Ann Bowling of University College London [16]. A shortened version of OPQoL-brief was later developed by Bowling et al. [6]. The OPQoL-brief consisted of 13 statements, with the participants being asked to indicate the extent to which they agree with each statement by selecting one of five possible options (“strongly disagree,” “disagree,” “neither agree nor disagree,” “agree,” and “strongly agree”). The range in the original version is based on the principle of point allocation (1–5). The items are summed to provide a total OPQoL-brief score. The total score of OPQoL-brief ranges from 13 to 65 and higher scores indicate better QoL. The OPQoL-brief questionnaire also includes a preliminary single item on global OoL. This single item is not scored with the OPQOL; it is coded as very good (5) to very bad (1). Bowling et al. [6] found a highly reliable and valid measure of QoL in old age in the OPQoL-brief scale.

Translation and linguistic validation in four phases: (1) translation, (2) reverse translation, (3) cognitive debriefing, and (4) proofreading. The OPQoL-brief was first translated by two local professional translators into Czech. Then, both translators and the local coordinator discussed the translation and created the first Czech version based on these two independently performed translations. Another professional translator then translated the OPQoL-brief back into English, and the local coordinator compared the reverse translation with the original English version. Any discrepancies were discussed between the translators, and a consensus was reached for the second version of the translation. Two translators and two experts from the field corrected the detected deviations. As a preliminary check, 20 Czech-speaking elderly people (mean age 71.2; 60% women) were then asked to read through the questionnaire with a research assistant and to indicate whether the instructions or any of the items were unclear. All items were deemed clear (cognitive debriefing). The proofreading was done by a proof-reader (native speaker). The final version was then created.

The following questionnaires were used to evaluate other parameters:

- *GDS-15* [22]. A Short Form of Geriatric Depression Scale consisting of 15 questions was developed in 1986 (response: yes/no). Scores of 0–4 are considered normal; 5–8 indicate mild depression, 9–11 indicate moderate depression, and 12–15 indicate severe depression. The Czech version was published by Jirák [23].
- *GAI* [24]. Geriatric Anxiety Inventory Scale consists of 20 “agree/disagree” items designed to assess common anxiety symptoms. A sum of these ratings composes a measure of general anxiety symptoms (ranging from 0 to 20), with higher scores indicating greater anxiety [24, 25].
- *SOC-13* [26]. The Sense of Coherence Scale is the short form of the SOC scale and consists of 13 items that comprise three components: comprehensibility (5 items), manageability (4 items), and meaningfulness (4 items). The respondents indicate whether they agree or disagree on a 7-category semantic differential scale with two anchoring responses tailored to the content of each item. The total score can range from 13 to 91, and a higher score indicates higher SOC.
- *RSES* [27]. Rosenberg Self-Esteem Scale is a 10-item Likert type scale, with items answered on a four-point scale: from strongly agree to strongly disagree.

### Data analysis

To test the psychometric properties of the questionnaire created, we tested the validity (construct validity, discriminant validity, convergent validity) and reliability (internal consistency, test–retest reliability). Also, the psychometric properties test sample size (≥ 500) was met, which can be considered very good [28]. The statistical program SPSS, v. 24.0 was used for data analysis.

### Structural validity

At first, we evaluated the single-dimensionality of the scale using the confirmatory factor analysis. The confirmatory factor analysis was performed using the robust maximum confidence value method (MLR), which corrects for abnormal distribution of items. The values of the parameters RMSEA (root mean square error of approximation), CFI (comparative fit index), TLI (Tucker-Lewis index), and SRMR (standardized root mean square residual) and GFI (Goodness of Fit Index) are given for individual models. CFI and TFI values should be close to 1.0 or at least exceed 0.90 [29]. The cut-off value for RMSEA is a recommended value of less than 0.06 [30] or with a strict limit of 0.07 [31]. The lower limit of the RMSEA confidence interval should be close to 0; the upper limit should not exceed 0.08 [31]. The SRMR value should be less than 0.05; however, a value under 0.08 is acceptable. The value acceptable for GFI is ≥ 90 [32]. The model showed borderline values, although the level of statistical significance of the chi-quadrate value was unsatisfactory. For this reason, we decided to perform an exploratory factor analysis, a principal component method, with Varimax rotation. It would help us better understand the factor structure of the OPQoL questionnaire. Prior to factor analysis, the suitability of factor analysis was verified using KMO (Kaiser–Meyer–Olkin measure) and Bartlett sphericity test. The model was tested as a single-factor model and subsequently with a value of 1.0 and greater.

### Convergent validity

Convergent validity was verified through Spearman’s correlation coefficient between the OPQoL-brief score and selected scales (GDS-15, GAI, SOC, RSES) and social support. We hypothesized that the QoL (OPQoL-brief) correlates negatively with anxiety [33] and depression [33–35] and correlates positively with sense of coherence [36, 37] and self-esteem [38]. Hendl [39] distinguishes the strength of the relationship association according to the value of the correlation coefficient “*r*” as follows: weak dependence (*r* = 0.1–0.3), medium dependence (*r* = 0.3–0.7), and strong dependence (*r* = 0.7–1). We supposed at least the medium correlation among the analyzed parameters. Correlation analysis between the selected parameters was performed because of the abnormal data distribution (Kolmogorov–Smirnov test) through Spearman’s correlation coefficient.

### Discriminant validity

Discriminant validity was assessed based on the OPQOL-brief ability to discriminate between healthy older individuals and older adults suffering from mental and physical illnesses. We hypothesized that the QoL of a person with mental/ physical illnesses was significantly different from that of a person without it [40]. The validity of the measure is supported if the mean of the QoL levels is significantly different between two groups. We tested the difference of the total score of QoL between two groups (older individuals with and without the illnesses) using the independent Wilcoxon test.

### Reliability

Internal consistency was determined through Cronbach’s alpha coefficient (*α*). The acceptable minimum value was set at *α* > 0.70 [41, 42]. Furthermore, we assessed the Cronbach’s alpha of domains without any items and the correlation of the individual items and the given domain (item-total correlation) with the acceptable minimum *r* > 0.40 [31].

To evaluate test–retest reliability, the ICC coefficient using two-way mixed model along with 95% confidence was computed. The coefficient of more than 0.70 was considered as excellent stability. Over a period of no longer than 5 days, the questionnaire was completed by 95 older adults to assess the test–retest reliability.

## Results

### Participant’s characteristics

The study involved 954 participants from 65 to 94 years of age, with an average age of 72 years. Almost three quarters of the participants were women (76.5%). Most participants lived in marriage (49.7%) and no longer worked (82.6%). A total of 866 (90.8%) seniors were treated for some chronic diseases on a regular basis. On average, an older adult was treated for 2.4 (SD = 1.6) diseases. The most common ones were cardiovascular (61.1%) and musculoskeletal system diseases (48.9%). The senior citizens also evaluated their subjective view of their health. The socio-demographic and health characteristics of the group are given in Table 1.

**Table 1:** Sociodemographic and health characteristics of sample (*n* = 954)

**Age**		
Mean (SD)	72.1	6.4
Min–max	65	94
**Gender ***N* (%)	***N***	**%**
Man	224	23.5
Women	730	76.5
**Marital status ***N* (%)		
Single	30	3.1
Married	474	49.7
Divorced	147	15.4
Widow	303	31.8
**Employment ***N* (%)		
Full time job	46	4.8
Part-time job	120	12.6
No job	788	82.6
**Living with**		
Alone	378	39.6
Spouse	468	49.1
Children	71	7.4
Another	37	3.9
**Illnesses**		
Cardiovascular (yes)	583	61.1
Oncological (yes)	75	7.9
Diabetic (yes)	186	19.5
Endocrinology (yes)	173	18.1
Respiratory (yes)	140	14.7
Gynecological (yes)	32	3.4
Urological (yes)	165	17.3
Sensory (yes)	275	28.8
Musculoskeletal (yes)	467	48.9
Neurological (yes)	99	10.4
Mental health problem (yes)	73	7.6
	**Mean**	**SD**
Number of disease/1 person	2.4	1.6
**Subjective health assessment**	***N***	**%**
Very good	19	2.0
Good	101	10.6
Fair	571	59.8
Poor	263	27.6

### OPQoL-brief items analysis

The average value of the overall QoL was found to be 3.79 (SD = 0.79). A total of 640 seniors (67.1%) considered their QoL to be good or very good. Only 50 (5.2%) seniors reported poor or very poor QoL. The average total score of all items was found at 54.49 (SD = 6.83). Descriptive statistics of individual questions is given in Table 2. The reliability of the questionnaire was found to be satisfactory (0.921). Table 2 also shows the Cronbach’s alpha if item deleted and item-total correlation values. A correlation exceeding 0.5 was determined for all items.

**Table 2:** The results for individual items of OPQOL-brief (*n* = 954)

	OPQOL-brief items	Mean	SD	Min–max	*α*^a^	I-T c
i1	I enjoy my life overall	3.91	0.78	1–5	0.913	0.705
i2	I look forward to things	4.03	0.76	1–5	0.913	0.701
I3	I am healthy enough to get out and about	4.22	0.80	1–5	0.916	0.640
I4	My family, friends, or neighbors would help me if needed	4.37	0.68	1–5	0.920	0.516
I5	I have social or leisure activities/hobbies I enjoy doing	4.09	0.82	1–5	0.912	0.734
I6	I try to stay involved with things	4.24	0.69	1–5	0.914	0.671
I7	I am healthy enough to have my independence	4.20	0.77	1–5	0.914	0.687
I8	I can please myself in what I do	4.17	0.71	1–5	0.911	0.760
I9	I feel safe where I live	4.19	0.76	1–5	0.918	0.571
I10	I get pleasure from my home	4.38	0.67	1–5	0.915	0.666
I11	I take life as it comes and make the best of things	4.37	0.62	1–5	0.914	0.709
I12	I feel lucky compared to most people	4.08	0.71	1–5	0.913	0.719
I13	I have enough money to pay for household bills	4.19	0.76	1–5	0.921	0.504
	**Total score**	**54.49**	**6.83**	**25–65**	**0.921**	**–-**

*SD* standard deviation, *I-T c* item-total correlation

^a^Cronbach’s alpha if item deleted

### Structural validity

First, we tested the single-factor model of OPQoL-brief on the first half of the sample using the confirmatory factor analysis. The model (13 items) gave significant *p*-values for all estimates, showing completely standardized factor loading from 0.44–0.58 and square multiple correlation (*R*^2^) ranging from 0.33 to 0.65. The model fit was as follows: *χ*^2^ = 242.84 (df = 55), *χ*^2^/df = 4.42, *p* < 0.001, CFI = 0.971, TLI = 0.959, RMSEA = 0.061 (95% CI = 0.053–0.069), SRMR = 0.034, and GFI = 0.960. The model showed borderline values, although the level of statistical significance of the chi-quadrate value was unsatisfactory. To better understand the factor structure, we performed an exploratory factor analysis on the second half of the sample. First, we evaluated the suitability of the factor analysis. The Kaiser–Meyer–Olkin measure was found to be adequate (0.935), exceeding the recommended minimum value of 0.60. Bartlett’s sphericity test also determined parameters of satisfactory values (A: *χ*^2^ = 6530.196; df = 78; *p* < 0.001).

**Fig. 1:**
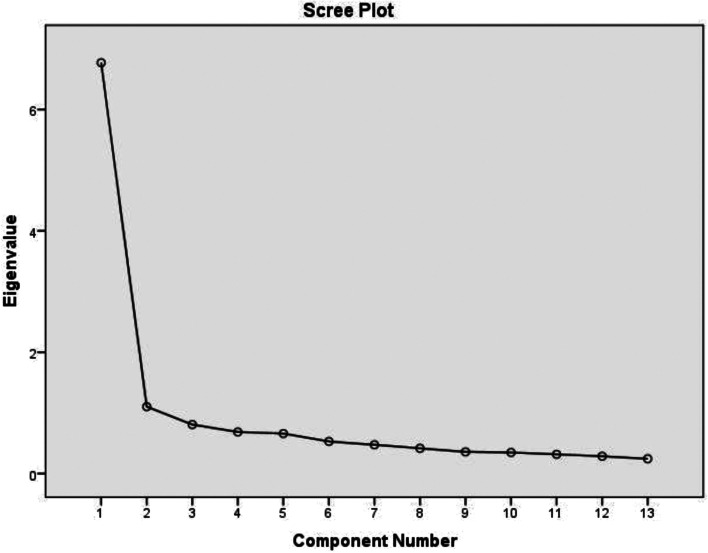
Scree plot of the 13 items OPQoL-brief questionnaire

The variability of the variables was explained by 52.1% through the factor analysis. First, a single-factor model was tested. Satisfactory factor loadings ≥ 0.55 were found for all thirteen items and excellent factor loading in 9 items (≥ 0.71). Subsequently, we tested model 2, which was extracted using eigenvalue 1.0 and greater (Fig. 1). A two-factor model was created. The first factor consisted of items focused on independence and active life (dom 1) and the second factor consisted of items focused on family and household (dom 2).

The results of the exploratory factor analysis for models 1 and 2 are shown in Table 3, where the values of loadings ≥ 0.45 are given. A satisfactory but lower α was found in the two-factor model than in the single-factor model.

**Table 3:** Exploratory factor analysis of the OPQoL-brief questionnaire

		**Model 1**	**Model 2**	
		Factor 1	Factor 1	Factor 2
i1	I enjoy my life overall	0.763	0.770	
i2	I look forward to things	0.762	0.768	
I3	I am healthy enough to get out and about	0.697	0.741	
I4	My family, friends, or neighbors would help me if needed	0.576		0.638
I5	I have social or leisure activities/hobbies I enjoy doing	0.790	0.721	
I6	I try to stay involved with things	0.731	0.696	
I7	I am healthy enough to have my independence	0.741	0.760	
I8	I can please myself in what I do	0.812	0.673	0.460
I9	I feel safe where I live	0.634		0.732
I10	I get pleasure from my home	0.722		0.791
I11	I take life as it comes and make the best of things	0.765	0.458	0.647
I12	I feel lucky compared to most people	0.773	0.530	0.571
I13	I have enough money to pay for household bills	0.564		0.636
	Cumulative % of total variance explained	52.08%	52.08%	8.48%
	Cronbach’s alpha (*α*)	0.921	0.916	0.747

Furthermore, correlation was made between the individual items. All correlations were significant at the significance level *p* < 0.001. For most items, a cross-correlation greater than 0.4 was found. Correlation analysis showed that OPQoL-brief items were highly inter-correlated (Table 4).

**Table 4:** Inter-item correlation of OPQoL-brief items

	I1	I2i	3i	4i	I5	I6	I7	I8	I9	I10	I11	I12
I1	1.000											
I2	0.672^a^	1.000										
I3	0.507^a^	0.495^a^	1.000									
I4	0.333^a^	0.353^a^	0.408^a^	1.000								
I5	0.625^a^	0.623^a^	0.530^a^	0.449^a^	1.000							
I6	0.495^a^	0.555^a^	0.455^a^	0.385^a^	0.616^a^	1.000						
I7	0.515^a^	0.516^a^	0.665^a^	0.401^a^	0.530^a^	0.566^a^	1.000					
I8	0.601^a^	0.610^a^	0.498^a^	0.424^a^	0.634^a^	0.610^a^	0.603^a^	1.000				
I9	0.384^a^	0.406^a^	0.394^a^	0.392^a^	0.447^a^	0.435^a^	0.447^a^	0.517^a^	1.000			
I10	0.454^a^	0.479^a^	0.404^a^	0.480^a^	0.516^a^	0.476^a^	0.467^a^	0.600^a^	0.641^a^	1.000		
I11	0.515^a^	0.516^a^	0.450^a^	0.558^a^	0.552^a^	0.526^a^	0.518^a^	0.595^a^	0.508^a^	0.641^a^	1.000	
I12	0.583^a^	0.553^a^	0.448^a^	0.402^a^	0.577^a^	0.492^a^	0.488^a^	0.589^a^	0.501^a^	0.548^a^	0.619^a^	1.000
I13	0.372^a^	0.351^a^	0.393^a^	0.369^a^	0.369^a^	0.392^a^	0.451^a^	0.417^a^	0.418^a^	0.466^a^	0.454^a^	0.500^a^

^a^Correlation is significant at the 0.01 level

A strong relationship was found between the overall OPQoL-brief score and the independence/active life (*r* = 0.948; *p* < 0.001) and family/household (*r* = 0.913; *p* < 0.001) domains. Also, strong relationship was determined between the mentioned domains to each other (*r* = 0.752; *p* < 0.001).

### Convergent validity

A negative correlation was found between the overall QoL, the total score of OPQoL-brief and both domains, and the scales measuring depression (GDS) and anxiety (GAI). A positive correlation was found between the overall QoL, the total score of OPQoL-brief, and the sense of coherence (SOC) and self-esteem (RSES); see Table 5.

**Table 5:** Correlation of QPQoL-brief and specific scales

	**Global QoL** (1 item)	**OPQoL-brief** (13 items)	**Independence/active life** (dom 1)	**Family/household** (dom 2)
GDS	− 0.490^b^	− 0.520^b^	− 0.549^b^	− 0.421^b^
GAI	− 0.339^b^	− 0.355^b^	− 0.348^b^	− 0.332^b^
SOC_total	0.416^b^	0.427^b^	0.417^b^	0.389^b^
SOC_C	0.331^b^	0.331^b^	0.312^b^	0.313^b^
SOC_MA	0.134^a^	0.105^a^	0.095^b^	0.106^b^
SOC_ME	0.431^b^	0.496^b^	0.486^b^	0.431^b^
RSES	0.370^b^	0.428^b^	0.418^b^	0.381^b^
Social support	− 0.260^b^	− 0.377^b^	− 0.318^b^	− 0.400^b^
Global QoL (1 item)	–	0.621^b^	0.631^b^	0.520^b^

*SOC_C* comprehensibility, *SOC_MA* manageability, *SOC_ME* meaningfulness, *RSES* Rosenberg self-esteem scale

^a^Correlation is significant at the 0.05 level

^b^Correlation is significant at the 0.01 level

### Discriminant validity

A positive correlation was found between the subjective health assessment and global QoL assessment (*r* = 0.441; *p* < 0.001), overall OPQoL-brief score (*r* = 0.425; *p* < 0.001), and independence/active life domains (*r* = 0.467; *p* < 0.001), and family/household (*r* = 0.467; *p* < 0.001).

As part of the discriminant validity assessment, we further compared the OPQoL-brief scores in people who are being treated for and not being treated for the disease. It was confirmed that the overall OPQoL-brief score and the domain “independence/active life” score are significantly higher in people without the disease compared to people with the disease (Table 6). The family/household domain did not show a lower QoL in people treated for respiratory, musculoskeletal, and neurological diseases.

**Table 6:** Comparison of the overall- and the domain score of OPQOL-brief in persons treated and not treated for physical or mental illness

**Disease**	**OPQoL-brief score**			**Independence/active life**			**Family/household**		
	Yes	No	*p*	Yes	No	*p*	Yes	No	*p*
Cardiovascular	53.81	55.38	**0.001**	28.43	29.92	**0.001**	21.21	22.19	**0.021**
Oncological	52.18	54.61	**0.004**	27.02	28.99	**0.001**	21.08	21.41	**0.184**
Diabetic	52.19	54.98	** < 0.000**	27.37	29.17	** < 0.000**	20.78	21.55	**0.001**
Respiratory	53.04	54.66	**0.010**	28.02	28.96	**0.012**	21.01	21.45	0.071
Gynecological	52.53	54.49	**0.022**	28.00	28.86	**0.049**	20.58	21.48	**0.046**
Urological	51.64	54.98	** < 0.000**	27.15	29.16	** < 0.000**	20.50	21.57	** < 0.000**
Sensory	53.02	54.99	** < 0.000**	27.96	29.16	**0.001**	20.95	21.57	**0.002**
Musculoskeletal	53.72	55.09	**0.004**	28.21	29.39	** < 0.000**	21.32	21.45	0.504
Neurological	51.82	54.71	**0.002**	26.66	29.06	** < 0.000**	20.93	21.44	0.201
Mental health	49.70	54.81	** < 0.000**	25.80	29.07	** < 0.000**	19.91	21.51	** < 0.000**

### Reliability

The internal consistency of the single-factor model of OPQoL-brief was found to be excellent *α* = 0.921 (Table 2). The ICC coefficient for the total score of the OPQoL-brief (ICC = 0.904; 95% CI: 0.677–0.959; *p* < 0.001) and global QoL (ICC = 0.899; 95% CI: 0.784–0.933; *p* < 0.001) suggests good test–retest reliability. Lower but satisfactory reliability was found for the domains of OPQoL-brief: Independence/active life: (ICC = 0.854; 95% CI: 0.583–0.913; *p* < 0.001) and family/household (ICC = 0.757; 95% CI: 0.381–0.886; *p* = 0.015).

## Discussion

Evaluating one’s QoL in old age has become an important part of the assessment of prevention measures and interventions provided to older adults in health and social services. Several questionnaires are recommended for the senior population. For easier administration, shorter versions of questionnaires are recommended for community-dwelling populations of older people, such as the OPQoL-brief [6] and the EQ-5D-3 instrument [10], for which satisfactory psychometric properties were determined [6, 43]. Longer versions of the questionnaires are available in the Czech version, namely OPQOL-35 and WHOQoL-old. For this reason, we decided to create a Czech version of the shorter questionnaire OPQoL-brief.

This article describes the psychometric properties of the Czech version of the OPQoL-brief scale on a cognitively normal community-dwelling elderly population. Our research confirmed the suitability of the single-factor model of the OPQoL-brief scale and the good psychometric properties of the scale. Good internal consistency of the scale was also found. Cronbach’s alpha measure of internal consistency exceeded the 0.70 threshold at 0.921 for 13 items. Inter-item reliability correlations for the OPQoL-brief were acceptable and ranged from 0.333–0.672. The ICC coefficient values (ICC = 0.904) were also satisfactory. Satisfactory reliability and internal consistency of the OPQoL-brief scale was found by Bowling et al. [6], but also Feizi and Heidari [40] in the Persian version and Caliskan et al. [35] in the Turkish version. Haugan et al. [33] found better reliability of the scale when excluding 5 items for a sample of seniors in nursing homes. They recommended using an 8-item scale for this group of old adults.

The suitability of the un-dimensional scale was confirmed in our research. Using the confirmatory factor analysis, we subsequently tested model 2, which was extracted using eigenvalue 1.0 and greater. A two-factor model was extracted. The first factor consisted of items focused on independence and active life, and the second factor consisted of items focused on family and household. The two-factor model showed acceptable but lower reliability and validity. Feizi and Heidari [40] present a three-factor model, namely socioeconomic well-being (5 items), emotional well-being (5 items), and physical well-being (4 items).

Convergent validity is defined as different methods measuring a construct give similar results [44]. In our study, correlation analysis was performed between OPQoL-brief and other scales (GDS, GAI, SOC-13, RSES, social support). We have chosen scales related to QoL evaluation. The correlation rate (*r* = 0.3–0.7) was found for all scales. Zielińska-Wieczkowska [37] stated that the sense of coherence is one of the crucial factors determining the life contentment and the ability to deal with difficult situations that are part of the process of aging. The meaningfulness of life is then an important component of motivation that stimulates the person to understand the world around them in a difficult situation that may be typical for older people. Individuals with strong SOC will be able to deal with various challenging life events. Zielińska-Wieczkowska [37] confirmed the correlation between the level of SOC in older adults with their QoL and depression.

Another finding of our research is the confirmed assumption of lower QoL in seniors treated for physical or mental illness compared to seniors who do not have this disease. This assumption was confirmed for the overall OPQoL-brief score and for domain 1: independence/active life. For the two family/household domain, this assumption has only been confirmed in some diseases.

## Conclusion

The results of our research showed that the shorter Czech version of the OPQoL-brief questionnaire has good reliability and validity and can be recommended for evaluating the QoL of seniors in a community within both health and social services. Completing the questionnaire is understandable for seniors and takes a maximum of 15 min. For evaluation, we recommend a single-factor model, which was found to have better validity and reliability.

## Data Availability

The Excel file can be provided on demand. RB (corresponding author) should be contacted by anyone requesting the data.

## Abbreviations

ASCOT: Adult Social Care Outcomes
CASP-19: Control, Autonomy, Self-Realization and Pleasure
CFI: Comparative fit index
EQOLI: Elderly Quality of Life Index
GAI: Geriatric Anxiety Inventory Scale
GDS: Geriatric Depression Scale
GFI: Goodness of Fit Index
HRQOL: Health related quality of life
OPQOL: The Older People Quality of Life
RMSEA: Root mean square error of approximation
RSES: Rosenberg Self-Esteem Scale
SD: Standard deviation
SOC: Sense of Coherence Scale
SRMR: Standardized root mean square residual
TLI: Tucker-Lewis Index
QoL: Quality of life
QUAL-E: Quality of life at the end of life
WHOQoL: World Health Organization Quality of Life

## References

[1] Czech Statistical Office [Český statistický úřad]. Population by age and marital status. [Obyvatelstvo podle věku a rodinného stavu]. In: Population development of the Czech Republic, population by age and marital status. [Vývoj obyvatelstva České republiky, Obyvatelstvo podle věku a rodinného stavu]. 2017. https://www.czso.cz/documents/10180/61565976/1300691801.pdf/1cce4610-d412-4d04-994e-dbf5a16386e4?version=1.2. Accessed 2017.

[2] Strategy of preparation for the aging of society 2019–2025. [Strategie přípravy na stárnutí společnosti 2019–2025]. In: Ministry of Labour and Social Affairs of Czech Republic. [Ministerstvo práce a sociálních věcí ČR]; 2019. http://amsp.cz/wp-content/uploads/2019/08/Strategie-p%C5%99%C3%ADpravy-na-st%C3%A1rnut%C3%AD-spole%C4%8Dnosti-2019-2025-ma_ALBSBADJYUA2.pdf. Accessed 6 June 2019.

[3] Dragomírecká E, Prajsová J. WHOQoL-OLD: user manual of the Czech version of the World Health Organization questionnaire for measuring the quality of life in old age [WHOQoL-OLD: příručka pro uživatele české verze dotazníku Světové zdravotnické organizace pro měření kvality života ve vyšším věku]. 1st ed. Prague: Psychiatrické centrum; 2009.

[4] World Health Organization (2012). WHOQoL user manual.

[5] Puvill T, Lindenberg J, De Craen AJ, Slaets JP (2016). Impact of physical and mental health on life satisfaction in old age: a population based observational study. BMC Geriatr.

[6] Bowling A, Hankins M, Windle G, Bilotta C, Grant R (2013). A short measure of quality of life in older age: the performance of the brief Olders Peoples of Life questionnaire. Arch Gerontol Geriatr.

[7] Bowling A. Psychometric properties of the older people’s quality of life quationnarie validity. Curr Gerontl Geriatr Res. 2009;2009:298950. 10.1155/2009/298950. 10.1155/2009/298950 PMC2819744 20168974

[8] Ware JE, Kosinski M, Keller SD (1994). SF-12 How to score the SF-12 physical and mental health summary scales.

[9] Ware JE, Snow KK, Kolinski M, Gandeck B (1993). SF-36 Health Survey manual and interpretation guide.

[10] EuroQol Research Foundation. EQ-5D-5L user Guide. In: EQ-5D user guides. 2019. https://euroqol.org/%20publications/user-guides/. Accessed Sept 2019.

[11] The WHOQOL Group (1998). Development of the World Health Organization WHOQoL-BREF quality of life assessment. Psychol Med.

[12] Brazier JE, Walters DJ, Nicholl JP, Kohler B (1996). Using the SF-36 and EuroQoL on an elderly population. Qual Life Res.

[13] CASP-19. Measuring quality of life in later life. https://casp19.com/background/. Accessed 15 Jan 2022.

[14] Steinhauser KE, Clipp EC, Bosworth HB, McNeilly M, Christakis NA, Voils CI, Tulsky JA (2004). Measuring quality of life at the end of life: validation of the QUAL-E. Palliat Support Care.

[15] Paschoal SM, Filho WJ, Litvoc J (2008). Development of elderly quality of life index-EQOLI: item reduction and distribution into dimensions. Clinics.

[16] Bowling A, Gabriel Z (2007). Lay theories of quality of life in older age. Aging Soc.

[17] Barkhausen T, Junius-Walker U, Hummers-Pradier E, Mueller CA, Theile G (2015). “It’s MAGIC”–development of a manageable geriatric assessment for general practice use. BMC Fam Pract.

[18] Dios-Quiroga F, Soliño-Lourido S, Pallas-Queijo C, González-Formoso C, Constenla-Castro A, Conde-Freire S, Clavería A (2020). Multidimensional geriatric assessment with MAGIC questionnaire and quality of life in elderly primary care patients. Int J Environ Res Public Health.

[19] Malley JN, Towers AM, Netten AP, Brazier JE, Forder JE, Flynn T (2012). An assessment of the construct validity of the ASCOT measure of social care-related quality of life with older people. Health Qual Life Outcomes.

[20] Bowling A, Stenner P (2011). Which measure of quality of life performs best in older age? A comparison of the OPQoL, CASP-19 and WHOQoL-OLS. J Epidemiol Community Health.

[21] Mareš J, Cígler H, Vachková E (2016). Czech version of OPQOL-35 questionnaire: the evaluation of the psychometric properties. Health Qual Life Outcomes.

[22] Yesavage JA, Brink TL, Rose TL, Lum O, Huang V, Adey MB, Leirer VO (1983). Development and validation of a geriatric depression screening scale: a preliminary report. J Psychiatr Res.

[23] Jirák R (2013). Gerontopsychiatrie (Gerontopsychiatry).

[24] Pachana N, Byrne G, Siddle H, Koloski N, Harley E, Arnold E (2007). Development and validation of the Geriatric Anxiety Inventory. Int Psychogeriatr.

[25] Heissler R, Kopeček M, Pachana NA, Franková V, Štěpánková GH (2018). Geriatric Anxiety Inventory (GAI) and its short form GAI-SF: Czech normative study. Cesk Psychol.

[26] Antonovsky A (1987). Unravelling the mystery of health. How people manage stress and stay well.

[27] Rosenberg M (1965). Society and the adolescent self-image.

[28] Boateng GO, Neilands TB, Frongillo EA, Melgar-Quiñonez HR, Young SL (2018). Best practices for developing and validating scales for health, social, and behavioral research: a primer. Front Public Health.

[29] Hooper D, Coughlan J, Mullen MR (2008). Structural equation modeling: guidelines for determining model fit. EJBRM.

[30] Hu L, Bentler PM (1999). Cutoff criteria for fit indexes in covariance structure analysis: conventional criteria versus new alternatives. Struct Equ Modeling.

[31] Ware JE, Gandek B (1998). Overview of the SF-36 Health Survey and the International Quality of Life Assessment (IQOLA) project. J Clin Epidemiol.

[32] Steiger JH (2007). Understanding the limitations of global fit assessment in structural equation modeling. Pers Individ Dif.

[33] Haugan G, Drageset J, André B, Kukulu K, Mugisha J, Utvaer BKS (2020). Assessing quality of life in older adults: psychometric properties of the OPQoL-brief questionnaire in a nursing home population. Health Qual Life Outcomes.

[34] Resende Rodrigues L, Santos Tavares D, Aparecida Dias F, Sousa Pegorari M, Fiori Marchiori G, dos Santos Tavares DM (2017). Quality of life of elderly people of the community and associated factors. J Nurs UFPE.

[35] Caliskan H, Aycicek GS, Ozurekci C, Dogrul RT, Balci C, Sumer F, Ozcan M, Karabulut E, Halil M, Cankurtaran M, Yavuz BB (2019). Turkish validation of a new scale from older peoples perspectives: older peoples quality of life-brief (OPQOL_brief). Arch Gerontol Geriatr.

[36] Gerasimčik-Plko V, Pileckaité-Markoviené M, Budotiené G, Ostapenko V (2009). Relationship between sense of coherence and quality of life in early stage breast cancer patients. Acta Med Lit.

[37] Zielińska-Wieczkowska H, Ciemnoczolowski W, Kedziora-Kornatowsk K, Muszalik M (2012). The sense of coherence (SOC) as an important determinant of life satisfaction, based on own research, and exemplified by the students of University of the Third Age (U3A). Arch Gerontol Geriatr.

[38] de Souza Júnior EV, Pires Cruz D, Reis Siqueira L (2022). Is self-esteem associated with the elderly person’s quality of life?. Rev Bras Enferm.

[39] Hendl J (2012). Přehled statistických metod zpracování dat: Analýza a metaanalýza dat.

[40] Feizi A, Heidari Z (2020). Persian version of the brief Older Peoples Quality of Life questionnaire (OPQoL-brief): the evaluation of the psychometric properties. Health Qual Life Outcomes.

[41] Terwee CB, Dekker FW, Wiersinga WM, Prummel MF, Bossuyt PM (2003). On assessing responsiveness of health-related quality of life instruments: guidelines for instrument evaluation. Qual Life Res.

[42] Peterson RA (1994). A meta-analysis of Cronbach’s coefficient alpha. J Consum Res.

[43] Kaambwa B, Gill L, McCaffrey N, Lanscar E, Cameron ID, Crotty M, Gray L, Ratcliffe J (2015). An empirical comparison of the OPQoL-Brief, EQ-5D-3 L and ASCOT in a community dwelling population of older people. Health Qual Life Outcomes.

[44] Polit DF, Beck CT (2012). Nursing research: generating and assessing evidence for nursing practice.

